# Understanding Neurodegeneration from a Clinical and Therapeutic Perspective in Early Diabetic Retinopathy

**DOI:** 10.3390/nu14040792

**Published:** 2022-02-14

**Authors:** Serena Fragiotta, Maria D. Pinazo-Durán, Gianluca Scuderi

**Affiliations:** 1 Ophthalmology Unit, Department NESMOS, S. Andrea Hospital, University of Rome “La Sapienza”, 00189 Rome, Italy; gianluca.scuderi@uniroma1.it; 2 Ophthalmology Unit, Department of Surgery, Faculty of Medicine and Odontology, University of Valencia, 46010 Valencia, Spain; dolores.pinazo@uv.es; 3 Ophthalmic Research Unit "Santiago Grisolía" Foundation for the Promotion of Health and Biomedical Research of Valencia FISABIO, 46017 Valencia, Spain; 4 Spanish Net of Ophthalmic Research “OFTARED” RD16/0008/0022, Institute of Health Carlos III, 28029 Madrid, Spain; 5 Spanish Net of Research in Inflammatory Diseases (REI), Cooperative Research Net Oriented to Results in Health (RICORS) RD21/0002/0032, Institute of Health Carlos III, 28029 Madrid, Spain

**Keywords:** diabetic retinopathy, neuroprotection, supplementation, aging, nutraceutics, diet

## Abstract

Recent evidence indicates that neurodegeneration is a critical element of diabetic retinopathy (DR) pathogenesis. The neuronal cells’ apoptosis contributes to microvascular impairment and blood–retinal barrier breakdown. Therefore, neurodegeneration represents an early intervention target to slow and prevent the development of microvascular alterations visible on clinical examination. Multimodal imaging features and functional assessment can permit the identification of neuronal damage in a subclinical stage before the recognition of DR signs. Clinical features of neurodegeneration are crucial in identifying patients at high risk of developing a vascular impairment and, thus, serve as outcome measures to understand the efficacy of supplementation. The optimal approach for targeting neurodegeneration contemplates the use of topical compounds that possibly act on different elements of the pathogenic cascade. To date, clinical trials available on humans tested three different topical agents, including brimonidine, somatostatin, and citicoline, with promising results.

## 1. Introduction

Diabetic retinopathy (DR) remains a critical global burden, with 103.12 million individuals affected and an estimated increase to 160.5 million by 2045 [1]. It represents a common and preventable complication of both type 1 and type 2 diabetes affecting the adult working population [2]. 

Screening and prevention are essential to successfully manage DR complications but challenging to achieve due to the significant disparity in resources across countries [3]. Microvascular changes on clinical examination represent the first recognizable clinical signs of DR, leading to the false conviction that DR is a mere microvascular disorder [4,5]. Nevertheless, increased neuronal cells’ apoptosis contributes to the microvascular impairment, affecting the glial cells that are mainly responsible for the maintenance of the integrity of the blood–retinal barrier [6,7]. Neural apoptosis starts early after diabetic onset with a constant and progressive rate of cellular death, involving retinal ganglion cells and other neural cells [6,8,9,10].

In clinical studies, the presence of neurodegeneration was confirmed by electroretinogram abnormalities, loss of dark adaption, reduced contrast vision, and color vision disturbances [11,12,13,14,15,16]. Beyond the functional implications, more recent studies demonstrated a thinning of the ganglion cell layer (GCL) and retinal fiber layer (RNFL) points toward an involvement of both axons and nerve bodies in DR [9,10,17,18,19,20]. 

Understanding the molecular mechanisms and the role of neurodegeneration is pivotal to identifying potential targets and therapeutical agents for an early intervention, slowing or even preventing the development of DR [5]. The mechanisms underlying neurodegeneration have been widely described, but the effectiveness and safety of neuroprotective substances in clinical trials deserve to be fully elucidated [21]. The present review provides a general overview of the molecular mechanisms and cellular interactions involved in DR neurodegeneration. However, the central core of the present work is to offer the latest insights and clinical evidence of neuroprotective agents tested on diabetic patients in clinical trials and to provide clinical and multimodal imaging features indicative for neurodegeneration in DR, useful as clinical outcomes in daily practice and future clinical trials. This approach would also introduce practical insights into the management of early DR from a clinical perspective.

## 2. Molecular and Cellular Interactions in Diabetic Retinopathy

### 2.1. Neurovascular Unit (NVU)

**Figure 1:**
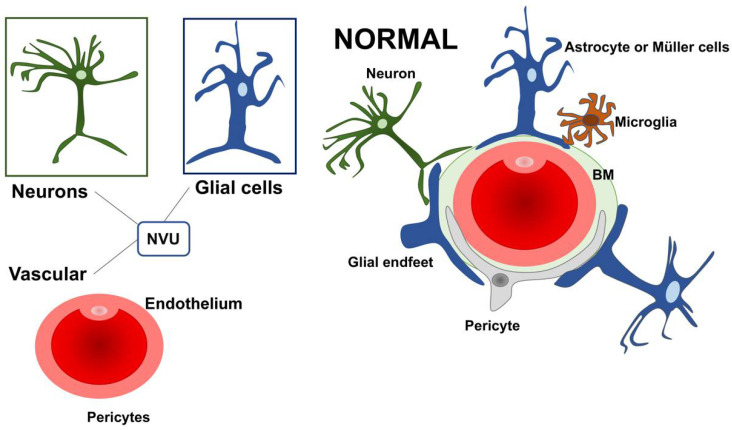
A schematic drawing of the neurovascular unit (NVU) in physiologic condition. The three main elements constituting the NVU are neurons, glial cells (astrocytes and Müller cells), and vascular components (endothelial cells and pericytes). NVU: Neurovascular Unit; BM: Basement membrane.

The retinal dysfunction in DR should be considered as a change in the retinal neurovascular unit [8]. The retinal neurovascular unit refers to the close interdependency among three main elements: neurons, glial cells (astrocytes and Müller cells), and vascular components (endothelial cells and pericytes) (Figure 1) [22]. Glial cells are strictly related to the neuronal homeostasis and neurotransmitter regulation. The interaction among the three elements (glial cells, neurons, and pericytes) promote the formation of the blood–retinal barrier (BRB) [8,23]. Retinal glial cells are elements that permit the communication between retinal blood vessels and neurons thanks to their arrangement and regulatory functions. In particular, the Müller cells present extensive arborization in direct contact with retinal neurons, participating in neurotransmission. They rapidly remove glutamate and γ-aminobutyric acid (GABA) in the inner retina, preventing neurotoxicity and reconverting glutamate into glutamine, providing a substrate for neurotransmitter synthesis [24,25,26,27,28,29,30].

Further, Müller cells participate in the “potassium spatial buffering”, a process dedicated to the redistribution and normalization of the K^+^ levels. This process is also essential to remove the fluid accumulation within the retina. The potassium is taken up by Müller cells in the extracellular space through Kir2.1 channels and deposited into the blood vessels using Kir4.1 channels [30,31,32,33,34]. The contribution of Müller cells in maintaining BRB seems to be also connected with the secretion of antiangiogenic factors, including pigment epithelium-derived factor (PEDF) and thrombospondin-1 [30]. Within the NVU, some cells can directly communicate with each other through a direct cell-to-cell interaction, as demonstrated for glial cells, endothelial cells, and pericytes. Other cellular subtypes talk at a distance via the secretion of ligands and/or exosomes.

### 2.2. Neurovascular Unit Impairment in Diabetes

**Figure 2:**
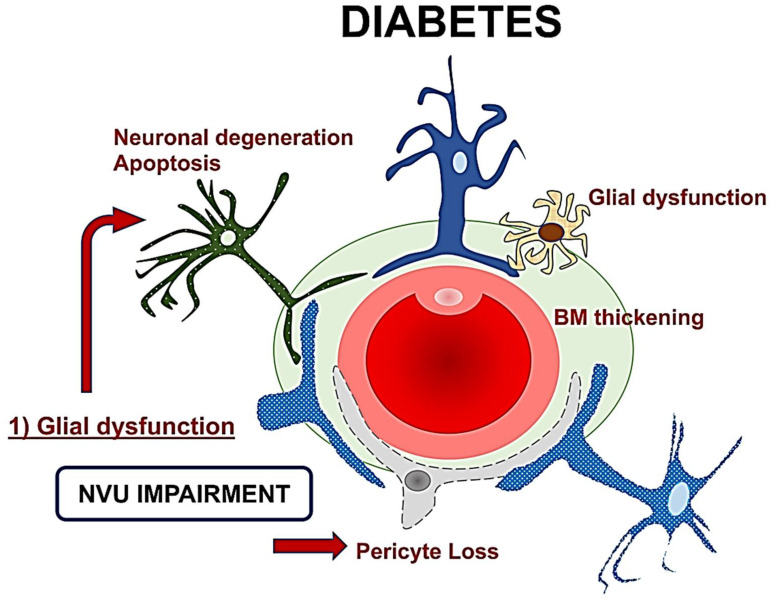
A schematic drawing of the neurovascular unit (NVU) in diabetes. The glial dysfunction appears to be the key element followed by neuronal degeneration, and then microvascular impairment with basement membrane thickening and pericytes loss. NVU: Neurovascular Unit; BM: Basement membrane.

The role of retinal glial cells can be considered central in the early stages of DR (Figure 2). In fact, perivascular microglial cells tended to grow in number and become hypertrophic within the innermost retinal layers, leading to an increased expression of glial fibrillary acidic protein (GFAP) in Müller cells and reduction in GFAP in astrocytes [35]. Activated glial cells progressed into an inflammatory state, producing several cytokines such as the interleukins (IL)-6 and -1b, TNF-a, and MCP-1. 

The resultant neuroinflammation can lead to cellular death and microvascular impairment [6,8]. The glial cells’ activation contributes to neuronal abnormalities, including neurite degeneration and neuronal apoptosis [8,22]. 

**Figure 3:**
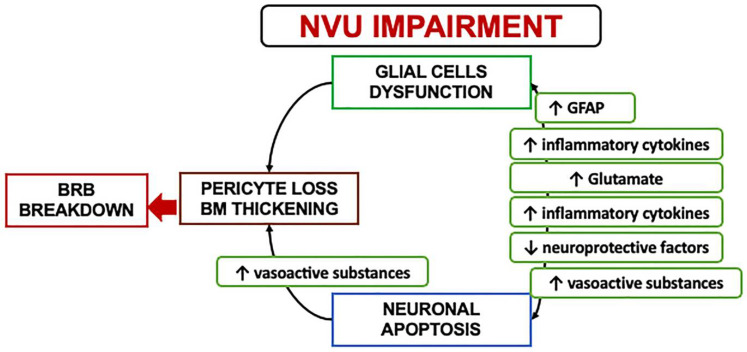
A schematization of the events characterizing the neurovascular unit impairment. NVU: Neurovascular Unit; BRB: Blood retinal barrier; GFAP: Glial fibrillary acidic protein.

In the early stages, a downregulation of the neuroprotective factors may be preponderant, including pigment epithelium-derived factor (PEDF), somatostatin (SST), and glucagon-like peptide 1 (GLP-1). The Müller cells dysfunction may further lead to an excess of glutamate, aggravating the neurotoxicity with neuron death [6,30,36,37,38,39]. Another consequence of Müller dysfunction is represented by the downregulation of the K+ channel subtype Kir4.1 and aquaporins but not Kir2.1, leading to an anomalous potassium intake and swelling [30,31,40]. The Müller glia are induced under hypoxic condition or glucose-deprived to increase their production and secretion of vascular endothelium growth factor (VEGF), which is also further increased by the altered permeability [23,40,41]. Glial activation and neural apoptosis represent the main mechanisms preceding the microvascular damage. Pericytes and an increased expression of the extracellular matrix components occurred before the involvement of the endothelial cells (Figure 3) [35,36].

### 2.3. Mechanisms and Clinical Implications of Neurodegeneration

The pathological hallmarks of neurodegeneration in diabetes include reactive gliosis, loss of neuronal functions, and neuronal apoptosis occurring before microangiopathy in experimental models [6,36,42,43]. NVU impairment is a critical event in the pathogenesis of the early stages of diabetes, and it is accompanied by an imbalance of several neuroprotective factors. Neurons are unable to proliferate or regenerate themselves, leading to a gradual and constant cell loss leading to chronic degeneration [36,44]. 

The potential loss of neuronal function in the absence of apoptosis can reasonably explain the early functional alterations seen in diabetic patients prone to developing diabetic retinopathy [12,37,38] Potential mechanisms involved in the decline of neuronal function may include the loss of synaptic proteins required for neurotransmission and intracellular calcium signaling alterations [38] It may be conceivable that other factors may be involved in the neurodegenerative process, including the protein misfolding altering endoplasmic reticulum homeostasis, but it has to be elucidated [45].

Retinal ganglion cells (RGCs) and amacrine cells have been identified as the first neurons involved in diabetes-induced apoptosis, but even photoreceptors may be affected. The direct consequence of an increased apoptotic death is a reduced thickness in the inner retinal layers and the nerve fiber layer clinically detectable through optical coherence tomography (OCT) [36]. The clinical recognition of a preclinical phase of retinopathy can be detrimental in identifying persons at risk for future retinal complications and visual loss, and, not less importantly, provide additional criteria for proper patient selection during clinical trials [46].

## 3. Clinical Hallmarks of Neurodegeneration in Diabetes

### 3.1. Functional Assessment

Early functional alterations in diabetes can be assessed using contrast sensitivity, dark adaption, frequency doubling technology perimetry (FDT), and multifocal electroretinogram (mfERG) [15,16,47,48,49]. 

Contrast sensitivity tested with the Pelli-Robson chart was significantly altered in eyes with diabetic retinopathy and correlated with the blood glucose levels [47]. Pelli-Robson contrast sensitivity was significantly reduced in diabetic patients without DR, with an average of 19% age-corrected reduction compared to healthy subjects [50]. Achromatic contrast sensitivity reduction was evident in diabetic patients independently from diabetic retinopathy microvascular complications, further supporting early neuronal involvement [51]. 

The functional integrity of the magnocellular component of the ganglion cells was estimated through Humphrey FDT technology. Type 1 diabetes patients presented an early functional impairment without any detectable microvascular alterations of diabetic retinopathy [49]. Diabetic patients without DR exhibited a mean of 2.9 dB loss compared to healthy subjects in the central region, while a more significant loss of sensitivity (3.93 dB) was evident in eyes with non-proliferative diabetic retinopathy [47]. FDT perimetry was superior to microperimetry in detecting a functional decline, more specific in tracing the neural damage of the RGCs [50]. A reduced dark adaption in diabetic patients without retinopathy or early changes indicated a rod cell impairment occurring as one of the earliest deficits in retinal function [48].

The most important functional factor predictive for the development of DR is represented by implicit time (IT) on mfERG. Patients with diabetes exhibited a significantly delayed P1 IT from rings 3 to 6, worsening in patients who presented microangiopathic abnormalities [52]. The clinical onset of DR intended as evidence of pathologic microvascular changes on clinical exam represents already a critical progression. The implicit time is spatially associated with retinopathy, correlated with the severity of the disease, and is a strong predictor for DR development over a short period. Thus, it is considered an essential surrogate outcome in clinical trials [11,12,13,53,54]. The mfERG P1 component is generated primarily by bipolar cells in the inner nuclear layer of the retina, further corroborating the presence of neural alterations in the absence of evident microvascular changes [12].

Recently, the photopic negative response (PhNR) has gained substantial interest in diabetic patients to estimate the RGCs response. It has been noted that the PhNR is reduced even in patients without clinically evident DR. These abnormalities can be detectable in people with diabetes with normal a- and b-waves on full-field ERG, indicating that the RGCs are affected independently from compromised photoreceptors or bipolar cells [55,56,57]. 

### 3.2. Structural Features on Retinal Imaging

In type 1 diabetes with no signs of diabetic retinopathy, the retinal nerve fiber layer (RNFL) imaged by GDx VCC was found to be significantly altered in eyes with HBA_1c_ ≥ 7% [49]. Preclinical retinal changes were demonstrated in the macular NFL, RGC layer (GCL), inner plexiform layer (IPL), inner nuclear layer (INL), and photoreceptors on OCT morphometric analysis in eyes with long-standing type 1 diabetes [58]. A significant difference in macular NFL was confirmed by van Dijk et al. [18] in patients with minimal or no signs of DR compared to healthy subjects. However, other studies did not show significant differences in macular NFL thickness between controls and diabetic eyes [59,60,61,62]. 

The alterations of the GCL are not of univocal interpretation. Some authors reported thickening of the GCL+IPL, hypothesizing a diffuse swelling occurring before the development of diabetic macular edema [58,63]. However, most studies reported a GCL+IPL thinning in diabetic patients [9,18,59,64,65]. In pediatric patients with DM1, the significant reduction in the ganglion cell complex (GCC) was postulated to be aggravated by hyperlipidemia, whereas the insulin demonstrated a possible protective effect with a thicker GCC [66]. Of note, the GCC is an automated OCT parameter that includes all three innermost retinal layers, RNFL, GCL, and IPL [67].

The INL/outer plexiform (OPL) thickening was attributed mostly to the change of the INL layer, which is essentially formed by the nuclei of bipolar and Müller cells and by the associations of horizontal and amacrine cells. This morphometric alteration may reflect a signature for Müller cells activation and hypertrophy due to swelling [64]. Similar results were achieved in a selected group of DM1 patients with a good glycemic control and no concomitant comorbidities. In this group, the INL was thicker in all the four explored quadrants (superior, nasal, temporal, and inferior) [68]. However, other authors obtained different results with a thinner INL in diabetic patients with no or initial signs of DR [58,69]. In general terms, the neurodegeneration characterizing early diabetes is accompanied by macular thickness reduction due to neural tissue loss. Still, as the disease progresses, the macular thickness tends to thicken for increased vascular permeability [69].

Recently, a cohort of patients with diabetes before any evident clinical signs of DR demonstrated a thinning of the inner retina (GCL-IPL). More interestingly, the structure–function relationship revealed that FDT and microperimetry measurements significantly correlated with structural parameters in the global and topographic analysis [70]. In DR patients with ETDRS level < 20, the presence of a GCL-IPL thinning was concomitant to mfERG abnormalities in 67% of cases. The lack of mfERG in the remaining 1/3 of the cases was explained by the fact that as the electric signal generated with mfERG is derived by bipolar cells and photoreceptors, thus, it may not reflect the status of all the cellular components of the inner retinal layers directly [52].

Glial cell proliferation in diabetic retinopathy has been hypothesized to be revealed through hyperreflective foci (HRF) visualization on OCT b-scan. A direct association between proinflammatory cytokines expressed by microglia, monocytes, macrophages and HRF localized in the inner retina has been established. The distinction between HRF of possible inflammatory origin and other etiologies includes the inner retina location, size ≤ 30 mm, absence of posterior shadowing, and reflectivity similar to the retinal fiber layer [71,72,73].

## 4. Clinical Trials and In Vivo Evidence Targeting Neurodegeneration

### 4.1. The Effect of Topical Neuroprotective Agents

The EUROCONDOR Study group was an international European consortium of leading experts from 11 European centers. This group conducted a prospective interventional phase II-III, randomized controlled clinical trial to evaluate the effect of topical (eye drops) neuroprotective agents on arresting or preventing neurodegeneration in early DR [NCT01726075] [52,74,75]. The population investigated included type 2 diabetes patients with a duration of at least five years and an ETDRS level of ≤35. Subjects were allocated in a 1:1:1 ratio to placebo, somatostatin 0.1%, or brimonidine tartrate 0.2% with the dosage of one drop b.i.d. in each eye. The randomization was based on a minimization algorithm that balanced the three groups according to the ETDRS level (<20 vs. 20–35).

From the selected population, 34.7% (156 patients) demonstrated mfERG abnormalities. Patients initiated with topical somatostatin or brimonidine presented a relatively unchanged mean IT after 24 months, compared to a placebo group that exhibited a significant IT decline. The mechanisms implicated in the arrest of neurodysfunction need to be fully elucidated. Brimonidine demonstrated an effect in promoting the survival and function of the retinal ganglion cells. Somatostatin is strongly downregulated in diabetic patients, and it is involved in a myriad of beneficial effects on the retina [74]. 

Topical administration of SST was found to be effective in preventing neurodegeneration in streptozotocin-induced diabetes mellitus rats treated for 15 days. The treatment with SST eye drops prevented b-wave ERG abnormalitis, glial activation assessed through GFAP expression, retinal cells apoptosis, and glutamate increase. Further, rats treated with SST presented an upregulation of GLAST protein levels with increasing levels compared to nondiabetic rats [76]. Experimental evidence on brimonidine suggested this as an ideal agent for neuroprotection, as confirmed by the presence of receptors in the retina and optic nerve, the adequate penetration into the vitreous and retina, and the induction of intracellular changes able to interrupt cellular apoptosis [77]. 

#### 4.1.1. Microvascular Changes Induced by Brimonidine and Somatostatin

The EUROCONDOR consortium investigated the effect of topical neuroprotection on the retinal vasculature analyzing the same subset of patients from the randomized clinical trial in two separate studies [75,78]. Retinal vessel caliber analysis was conducted using a semiautomatic software VAN, Department of Ophthalmology Visual Science, University of Wisconsin, Madison, WI, USA, which was widely validated to measure the largest six arterioles and venules around the optic nerve head 0.5–1.0 disc diameters from the disc margin [79,80,81,82,83,84]. Retinal vessel caliber was associated with the structural alterations of retinal neurodegeneration. In detail, the macular GCL thickness was associated directly with the arteriolar caliber. In contrast, the retinal venular caliber was negatively related to macular retinal thickness but directly correlated to peripapillary RNFL thickness. The authors hypothesized that retinal venular caliber could be connected to impaired vascular autoregulation and ischemia or systemic blood pressure changes. However, the direct association between central arteriolar caliber and macular RGCs/GCL can link microvascular impairment and neurodegeneration in early diabetic eyes [75].

More interestingly, the ensuing study investigated the role of brimonidine and somatostatin topical therapy on retinal vascular dilatation. The results demonstrated that only patients with a preexisting DR developed a significant arteriolar and venular widening after topical administration of the neuroprotective agents. No substantial differences were noted between the brimonidine and somatostatin groups, but the vasodilatation was significant in the treated groups compared to the placebo group. The only factor that seems to be involved in more considerable vasodilatation was represented by the staging of DR. Patients with mild NPDR in the treated groups exhibited a venular dilatation of more than three times greater than the placebo group. The venular dilatation induced by topical neuroprotective agents was between 13.9 and 14.3 mm. These findings were explained by hypothesizing that the neuroprotective agents may have had a favorable effect on the NVU impairment, thus resulting in improved vascular autoregulation and vasodilatation [78].

Systemic adverse effects were similar between the brimonidine (14%) and placebo group (14%) and even lower in the somatostatin group (8%). This finding confirmed a safe systemic profile of the topical agents administered. Among ocular or local adverse effects, ocular hyperemia and eye pain were more commonly detected in the brimonidine subgroup [74].

#### 4.1.2. Serum Biomarkers and Retinal Neurodysfunction

An ancillary study from EUROCONDOR [85] aimed to test some circulating molecules in predicting the worsening of retinal neurodysfunction. The selected molecules reflected potential biomarkers for neurodysfunction based on the NVU impairment pathogenic mechanisms. The mechanisms included basement membrane thickening, the accumulation of advanced glycation end-products (AGEs), and oxidative stress. The circulating biomarkers tested included laminin P1, reflecting the increased thickening and turnover of the basement membrane, and N-epsilon-carboxy methyl lysine (CML), which is an AGEs involved in the early DR stages. Serum asymmetric dimethylarginine (ADMA) was used as a surrogate marker of oxidative stress, and more specifically, endothelium-derived oxidative stress. 

A direct correlation between IT and CML levels was found at baseline. An increasing CML concentration was associated with IT decline over time in the placebo group but not in the groups treated with topical brimonidine or somatostatin. The levels of CML correlated with macular thickness and GCL-IPL. Baseline Lam-P1 levels were associated with GCL-IPL and increased macular thickness at the end of the followup in the placebo group but not in the treatment groups. Of note, a significant decrease in Lam-P1 was observed in the group treated with somatostatin at 6 and 12 months. 

The authors concluded that the decline in IT associated with an increasing CML concentration over time observed in the placebo group represented progressive neurodegeneration reflecting the natural history of the disease. However, the use of topical brimonidine or somatostatin prevented the decline in IT and the increasing CML levels, further corroborating their utility as neuroprotective agents. Another important finding is represented by a significative downregulation of Lam-P1 levels in patients treated with SST, suggesting a direct effect of SST on basement membrane protection. Both CML and LamP1 could be helpful biomarkers in early DR. 

### 4.2. Topical Citicoline

Citicoline, also known as Cytidine 5′-diphosphocholine, has been widely studied in neurodegenerative disorders. The effects of citicoline in diabetes appeared to be directed to a multitude of mechanisms, including glutamate excitotoxicity and oxidative stress modulation, increasing neurotrophin levels, improving the release of the neurotransmitters, enhancing the axonal transport and neuron homeostasis, promoting mitochondrial function, and modulating insulin signaling. Citicoline is involved in the sphingomyelin biosynthesis favoring the plasma membrane stabilization in the RGC axons [86,87].

The first randomized clinical trial [NCT04009980] testing the efficacy of citicoline eyes drops was conducted on patients with type diabetes 1 and mild signs of NPDR during three years of followup. Patients were randomized into treatment groups using citicoline and vitamin B12 eye drops (OMK2^®^ containing citicoline 2%, hyaluronic acid 0.2%, and cyanocobalamin 0.05%; Omikron Italia srl, Italy) 1 drop t.i.d and a placebo consisting of eye drops containing hypromellose 0.3%, 1 drop t.i.d. Patients were followed at 12, 24, and 36 months performing 24-2 and 10-2 FDT, OCT with Spectralis mapping software, segmentation software, and OCT angiography to assess the superficial and deep vascular plexus. Adaptive optics was also performed sing Rtx1 (Imagine Eyes, Orsay, France) [88]. 

At the end of the followup, the placebo group demonstrated significant worsening on 10-2 mean sensitivity, not evident in the treated group. The reduction in mean sensitivity was hypothesized to result from a higher susceptibility of the magnocellular RGCs to hyperglycemia. On a morphological evaluation, subjects who received citicoline treatment maintained the INL and OPL thickness over time, while the placebo group showed a significant INL increase and OPL decrease. These anatomical changes were explained with Müller cells activation preceding the BRB breakdown. The vascular analysis on OCTA added intriguing insights, as the placebo group demonstrated a progressive rarefaction of the vessel density on both superficial and deep vascular plexus. These findings further suggest the protective role of citicoline on the microvascular impairment in DR. 

#### The Effect of Citicoline on Retinal Function

An ancillary outcome study of the original clinical trial testing citicoline eye drops [NCT04009980] reported the results from mfERG evaluations on the same subset of patients. Although the groups did not differ from each other at baseline, the treated group demonstrated a large percentage of eyes with increased averaged response amplitude densities (RAD) values. The treatment arm presented increased RAD values in 75% for R2 and R1+R2+R3 and 62.5% for R1 and R3, while the placebo group demonstrated a significant reduction in RAD values of 90% for R1, 70% for R2, and 80% for R3 and R1+R2+R3. The RAD values were also significantly different between the groups at 36 months, where a significant decrease in mfERG RADs was evident in the 0–10 central retinal degrees in the placebo group. By contrast, the group treated with citicoline exhibited a significant increase in RADs evaluated at 0–10 central degrees. The authors explained the results indicating that citicoline and vitamin B12 eye drops may improve the function of preganglionic elements, including photoreceptors and bipolar cells, located in the 10 central degrees [88]. 

## 5. Conclusions and Future Perspectives

Diabetic retinopathy has been considered for decades as a mere microvascular disease. However, in the early stages of the disease, neurodegeneration predominates, preceding the microvascular damage. Therefore, early intervention should target NVU and neurodegeneration first to block the pathogenic cascade leading to microvascular complications. The main limitation when approaching diabetic complications from an NVU point of view is that several pathways are involved in neuronal cell death, thus, implying a combination of neuroprotective agents to inhibit or prevent the disease progression effectively. Another critical limitation is that neuronal regeneration is not achievable under normal conditions. Once axonal degeneration occurs, it leads to an irreversible cellular loss [22]. Topical treatments are preferable when approaching diabetes to avoid systemic interactions. Both topical brimonidine and somatostatin demonstrated an effect on the neurovascular components in patients with preexisting mild non-proliferative diabetic retinopathy. The topical administration of somatostatin or brimonidine was able to arrest the progression of the implicit time on mfERG over two years of followup, confirming a protective effect in preventing the worsening of neurodysfunction [52]. This neuroprotective effect seems to modulate the microvascular response on type 2 diabetes, inducing retinal vascular dilatation. The arterioral and venular dilation after administration of topical neuroprotective agents occurred only in eyes with preexisting mild NPDR, further confirming the rationale in targeting neuroprotection early to delay diabetic retinopathy [78]. The use of topical citicoline combined with vitamin B-12 indicated a functional and morphological effect on retinal ganglion cells, presumably due to neuroenhancement, neuroprotection, and the neuroregeneration properties of citicoline. Patients under citicoline topical treatment preserved superficial and deep retinal vascular parameters on OCTA, indicating a potential protective effect on microvascular impairment over time [88,89]. More importantly, all these neuroprotective compounds are safe on a systemic profile with no potential interactions or side effects, except for ocular hyperemia as the most frequent local effect in the brimonidine group [78].

Recently, novel pharmacological interventions demonstrated promising outcomes in experimental settings. In particular, glial cell activation inhibitors (e.g., topical bosentan, glucagon-like peptide-1, and suppressors of cytokine signaling (SOCS) proteins), and GSK-3β-mediated RGC synaptic neurodegeneration inhibition [5]. The neuroprotective role of heat shock proteins has been observed in several models of retinal neurodegeneration. One molecule is represented by arimoclomol, which upregulates heat shock proteins protecting against neurodegeneration in amyotrophic lateral sclerosis and retinitis pigmentosa [90]. Furthermore, the oxidative stress contributing to neurodegeneration can also be targeted using antioxidants and micronutrient supplementation [91,92]. The use of nutritional supplements containing antioxidants and omega 3 fatty acids in type 2 diabetes significantly reduced the oxidative load, increasing circulating antioxidants [93]. Despite the growing interest in antioxidant supplementation in DR, the findings are promising but heterogeneous in terms of molecules tested, doses, and results, complicating the interpretation of the clinical outcomes. [93,94,95,96,97,98]. 

This narrative review was meant to bring practical recommendations and evidence from clinical studies, and thus the details on experimental findings are beyond the scopes of the present work. However, it is crucial to understand the current clinical approach to finding potential future directions. 

With more sophisticated imaging technologies, recognizing neurodegenerative damage has become possible by evaluating both functional and morphometric features. The prompt recognition of neurodegenerative changes in a preclinical status can be detrimental to driving the clinical approach and avoiding microvascular complications. Further, the distinction of functional and morphological surrogate biomarkers of neurodegeneration can be particularly beneficial for future studies and clinical trials. Several substances demonstrated promising experimental results that need to be tested alone or combined to contrast neurodegenerative early damage in DR in vivo. 

## Data Availability

Not applicable.

## Abbreviations

AGEs	advanced glycation end-products
BRB	blood-retinal barrier
DR	diabetic retinopathy
FDT	frequency doubling technology perimetry
GCL	ganglion cell layer
INL	inner nuclear layer
I	inner plexiform layer
PL	hyperreflective foci
HRF	implicit time
IT	multifocal electroretinogram
mfERG	optical coherence tomography
OCT	optical coherence tomography angiography
OCTA	outer plexiform layer
OPL	neurovascular unit
NVU	retinal ganglion cell layer
RGCs	retinal fiber layer
